# Supplementary material for: Novel Driver Strength Index highlights important cancer genes in TCGA PanCanAtlas patients
Source: PeerJ. 2022 Aug 11;10:e13860. doi: 10.7717/peerj.13860 (PMC9375969; doi:10.7717/peerj.13860)
Supplement: Supplemental Information 2 [file peerj-10-13860-s002.zip › COHORTS/genes plots/age/2021_8_16_14_9_distribution_cohorts_males_35-39.pdf]

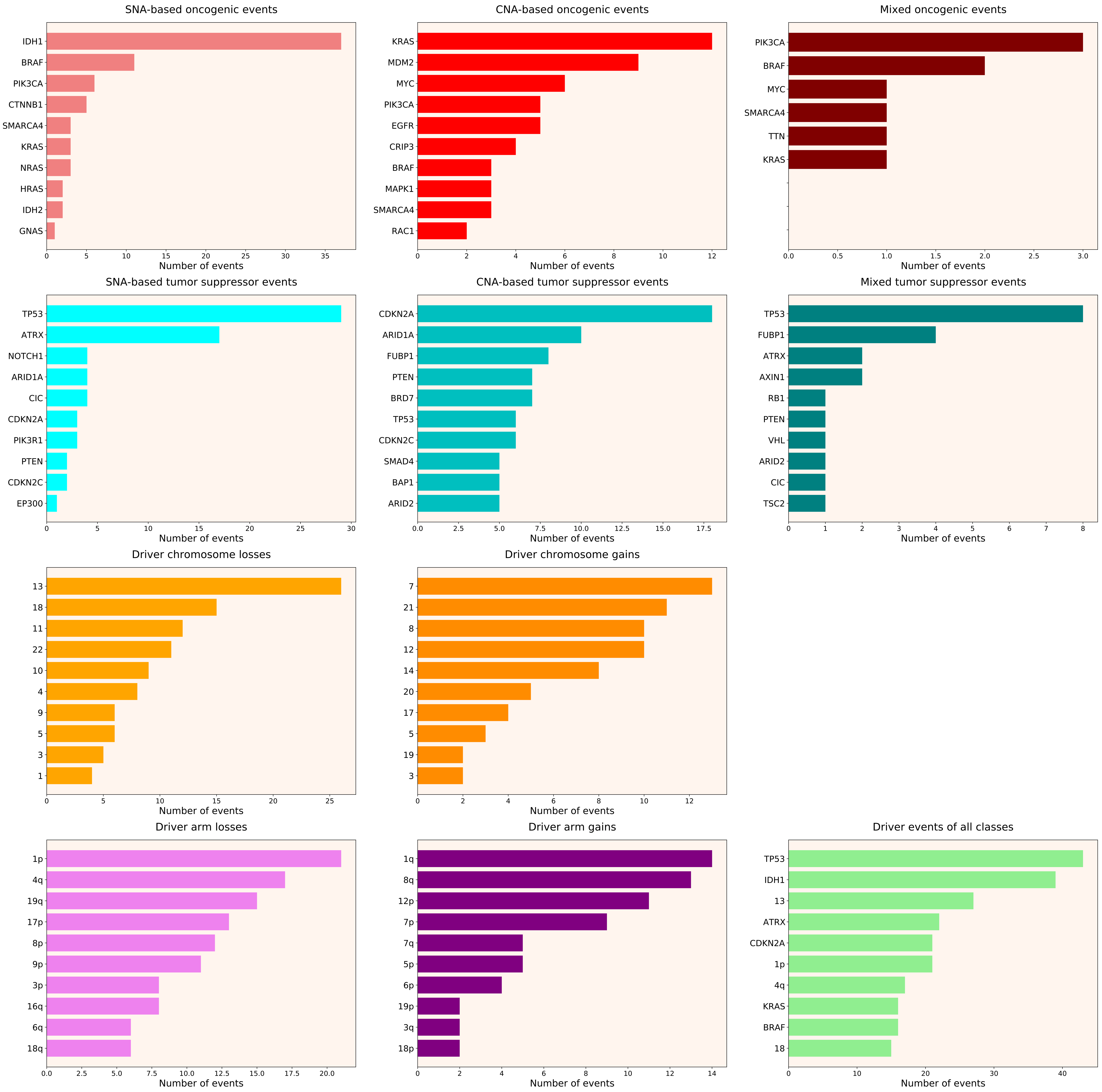
SNA-based oncogenic events
IDH1
BRAF
PIK3CA
CTNNB1
SMARCA4
KRAS
NRAS
HRAS
IDH2
GNAS
Number of events
CNA-based oncogenic events
KRAS
MDM2
MYC
PIK3CA
EGFR
CRIP3
BRAF
MAPK1
SMARCA4
RAC1
Number of events
Mixed oncogenic events
PIK3CA
BRAF
MYC
SMARCA4
TTN
KRAS
Number of events
SNA-based tumor suppressor events
TP53
ATRX
NOTCH1
ARID1A
CIC
CDKN2A
PIK3R1
PTEN
CDKN2C
EP300
Number of events
CNA-based tumor suppressor events
CDKN2A
ARID1A
FUBP1
PTEN
BRD7
TP53
CDKN2C
SMAD4
BAP1
ARID2
Number of events
Mixed tumor suppressor events
TP53
FUBP1
ATRX
AXIN1
RB1
PTEN
VHL
ARID2
CIC
TSC2
Number of events
Driver chromosome losses
13
18
11
22
10
4
9
5
3
1
Number of events
Driver chromosome gains
7
21
8
12
14
20
17
5
19
3
Number of events
Driver arm losses
1p
4q
19q
17p
8p
9p
3p
16q
6q
18q
Number of events
Driver arm gains
1q
8q
12p
7p
7q
5p
6p
19p
3q
18p
Number of events
Driver events of all classes
TP53
IDH1
13
ATRX
CDKN2A
1p
4q
KRAS
BRAF
18
Number of events
